# Among-individual asynchrony but not genetic diversity is associated with temporal stability of tree growth in natural *Quercus robur* oak stands

**DOI:** 10.1098/rsbl.2025.0180

**Published:** 2025-09-10

**Authors:** Marcus Hall, Johanna Sunde, Markus Franzén, Anders Forsman

**Affiliations:** ^1^ Department of Biology and Environmental Science, Linnaeus University, Kalmar, Kalmar County, Sweden; ^2^ Department of Forestry and Wood Technology, Linnaeus University, Kalmar, Sweden; ^3^ Department of Physics, Chemistry and Biology, Linköping University, Linköping, Sweden

**Keywords:** ecosystem stability, genetic variation, synchrony, productivity, temporal stability, biodiversity–ecosystem functioning

## Abstract

Theory, manipulation experiments and observational studies on biodiversity and ecosystem functioning largely concur that higher intraspecific diversity may increase the overall productivity of populations, buffer against environmental change and stabilize long-term productivity. However, evidence comes primarily from small and short-lived organisms. We tested for effects of genetic diversity on variation in forest growth by combining long-term data on annual individual growth rate (basal area increment (BAI)) with estimates of intrapopulation genetic variation (based on RAD-seq SNPs) for 18 natural *Quercus robur* pedunculate oak populations. Higher total or adaptive genetic variability of populations was neither associated with faster average growth nor with increased temporal or spatial stability of growth nor with among-individual asynchrony in growth. However, as expected, we found that greater asynchrony of growth responses within the populations increased their temporal stability. Together, these findings point towards a negligible role of genetic variation in structuring growth patterns in natural populations of tree species. Identifying which environmental factors and phenotypic traits (and its genetic basis) contribute to asynchronous growth responses is an important next step towards a better mechanistic understanding of the causes of temporal stability in tree growth and forest productivity.

## 1. Introduction

Genetic and phenotypic diversity are key components of biodiversity, and the extent to which individuals differ within a population can have far-reaching evolutionary and ecological consequences [1–4]. Theory, manipulation experiments and observational studies have highlighted that greater among individual variability and asynchronous non-correlated responses could increase the overall performance and productivity of populations, buffer against environmental fluctuations, and thereby stabilize populations and long-term productivity [2,3,5–7]. It is also conceivable that diversity has no influence on the average performance of the population but instead reduces the variance in performance, either over time within populations in changing environments or among populations that inhabit different environments [3,8–14].

Several mechanisms, operating on different temporal and spatial scales, have been proposed to contribute to the improved performance of more diverse populations. These include, but are not limited to: *sampling effects* meaning a higher probability that more diverse groups include preadapted phenotypes; *niche complementarity effects* when different genotypes and phenotypes utilize and can withstand different spectra of the environment, resulting in reduced competition, more efficient exploitation of available resources and improved tolerance to a broader range of conditions in more diverse groups; *facilitation* when the presence of one genotype or phenotype promotes the success of others; *enemy protection/protective polymorphism/associational resistance* when greater diversity reduces susceptibility to pathogens, parasites, predators or herbivores; and *evolvability and evolutionary rescue*, whereby heritable individual variation enables faster adaptation to changing conditions; all of which have been reviewed and discussed in detail elsewhere [2,3,5–7,15]. These drivers, sometimes referred to using different terminology, are not mutually exclusive, several processes may be simultaneously involved, and their relative importance likely varies according to species and environmental settings.

Experimental support for positive effects of higher interindividual diversity mainly comes from short-lived organisms (e.g. arthropods, fish, birds, herbaceous plants) and plant species capable of clonal reproduction (e.g. *Spartina alterniflora, Zostera marina*) [3,7,16–18]. A meta-analysis has also shown that positive effects of diversity manifest more strongly under complex natural conditions in the wild than under semi-natural and standardized laboratory conditions [17], likely reflecting that a larger number of the many ways by which variation can promote performance come into play under more stressful and complex environmental conditions [3]. Accordingly, intraspecific diversity effects may be of particular importance in temperate and boreal forests at higher latitudes, where environmental conditions are harsh, tree species are few and trees have high genetic and phenotypic diversity [19–21]. However, compared with the rich literature on the consequences of species diversity for the stability, functioning and resilience of forest ecosystems [22–28], the effects of intraspecific genetic diversity on productivity in natural forest stands remain largely unexplored (but see [29–32] examining the effects of genetic diversity on productivity in experimental forest stands).

Intraspecific trait variation of potential importance for growth performance of tree species in temperate forests, here exemplified by the pedunculate oak *Quercus robur*, include but are not limited to the timing of budburst and autumn leaf senescence [33–36], growth–herbivore defence trade-offs [37] and water use efficiency [38]. Assuming that such functionally important traits have a genetic basis, the overall genetic diversity and differentiation among individuals within populations may be used as a predictor of variation in growth performance within and among populations.

We investigated whether and how the degree of genetic diversity among individuals within oak (*Q. robur*) stands was associated with the average annual growth rate, the temporal (among years) or spatial (among trees) stability in growth rate or the among-individual asynchrony in growth rate. Finally, we examined whether greater asynchrony of individual growth responses was associated with temporal stability of the population.

## 2. Methods

### (a) Study system and sampling

The pedunculate oak (*Q. robur*) is a deciduous tree species with a distribution range spanning most of Europe and with its eastern range limit at the Ural Mountains [39]. It hybridizes with closely related oak species [40], and exhibits a high degree of genetic and phenotypic variation within populations [41]. As such, *Q. robur* is well suited for examining the role of genetic diversity for the overall performance and stability of growth rates of natural populations.

**Figure 1.**
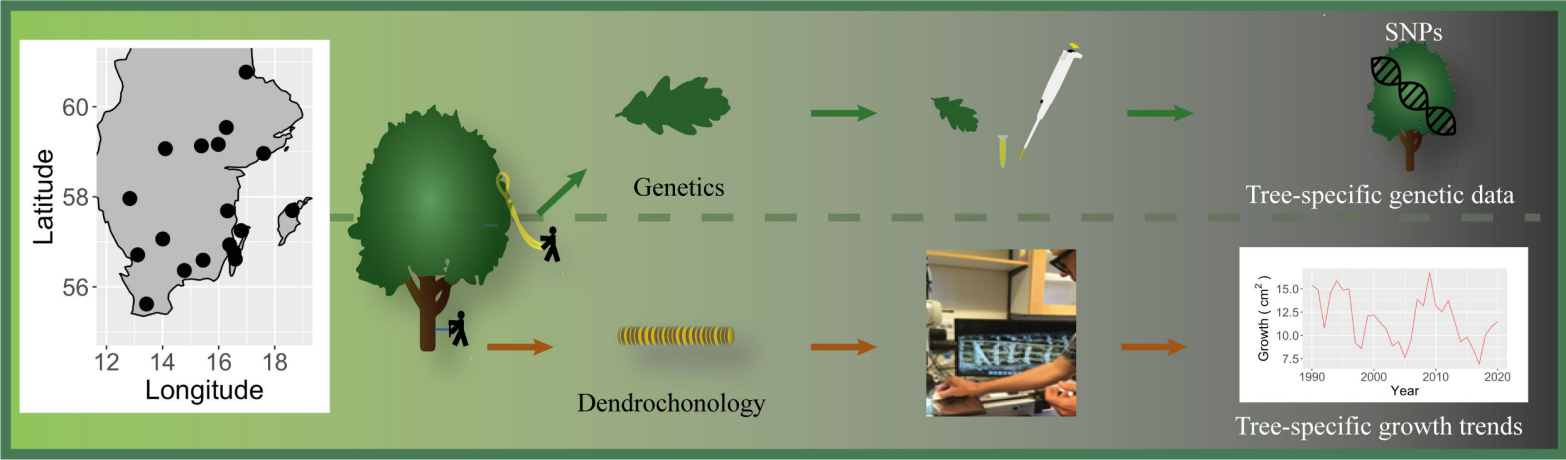
Location of the oak populations in southern Sweden and a schematic presentation of the sampling and data processing. At each location, we sampled 10 oak trees, collecting individual-specific information on the trees genetic make-up (upper panel) and on its annual growth rate and how it fluctuates over time (tree ring data—bottom panel).

We collected tree core samples (for growth estimates) from 18 oak dominated (oak >50% of the trees) forest stands distributed across southern Sweden (age of the sampled trees across stands in 2005, 81 ± 33.7 years; size (DBH) 40.6 ± 7.57 cm; stand basal area 26.9 ± 7.57 m^2^ ha^−1^, mean and s.d.), sampling 10 trees per population for growth estimates [42] (table 1). We only included oaks that were part of the main forest canopy (i.e. not trees from the understory/secondary tree layer) in the sampling. The trees were selected to capture the size distribution of the dominant oaks within the site, and such that they were evenly distributed across the stand. To estimate the degree of genetic variability among individuals within the populations, we also collected leaves for genotyping with restriction site-associated DNA sequencing (RADseq) from each tree sampled for growth estimates (subset of the samples used in [43]) (Figure 1). Based on a combination of leaf morphology and molecular markers, Hall *et al.* [43] found that some (*n* = 5) of the populations in this study include *Quercus petraea* and putative *Q. petraea* × *Q. robur* hybrid trees. We therefore excluded the *Q. petraea* (*n* = 9) and hybrids (*n* = 3) from the subsequent analysis.

**Table 1.** Information on the average annual growth rate (BAI) and the temporal and spatial stability of the sampled populations. The genetic variability of the populations for all loci and the putatively adaptive loci. Stand-specific information on the average size (diameter) and age of the sampled trees at the location, and the average stand basal area (m^2^ ha^−1^) at the location.

	growth			genetic variability		stand information		
population	BAI (cm^2^)	temporal stability	spatial stability	all loci	putative adaptive l	tree size DBH (cm)	tree age (years)	stand basal area (m^2^ ha^−1^)
Aga	9.55	4.62	1.87	0.50	0.54	27.8	62.3	20.9
Ank	15.05	3.93	2.13	0.52	0.54	36.5	85.6	17.8
BJ	3.99	4.50	2.26	0.50	0.22	27.6	174.0	23.5
Bjo	20.99	6.38	2.21	0.57	1.21	45.2	57.2	29.2
Fh	9.59	5.06	2.27	0.51	0.39	43.1	105.8	42.1
Gar	13.05	6.52	1.76	0.50	0.42	35.9	53.0	25.8
Lan	16.79	6.93	2.30	0.50	0.38	57.2	119.2	27.9
Ral	11.85	6.84	2.01	0.49	0.29	43.2	75.6	32.2
Sth	18.16	2.93	2.16	0.48	0.49	33.8	43.5	18.2
Str	20.62	3.21	1.97	0.53	0.36	40.2	61.3	20.6
Tanr	20.66	4.44	1.63	0.50	0.30	37.6	60.6	30.7
Tes	9.24	3.86	1.53	0.47	0.50	40.3	108.1	41
Trao	13.00	2.30	1.63	0.49	0.33	33.1	57.6	18
Vag	13.53	2.87	1.89	0.51	0.33	33.2	39.9	32.8
Var	18.62	6.79	2.29	0.50	0.31	46.4	79.8	35.5
Vas	18.57	3.04	1.91	0.51	0.45	41.5	63.4	21.1
VasV	16.59	3.28	1.64	0.52	0.48	54.6	115.7	25.6
Vin	23.77	3.73	2.38	0.45	0.33	52.7	94.4	21.9

### (b) Estimating tree growth

We used the collected pith to core wood samples for the 10 oak individuals per population to determine tree age, estimate the annual BAI (growth rates) of individuals and populations and how they varied across time: detailed methods for estimation of annual tree-ring widths as described in [42,44] (figure 1). For annual growth estimates, we converted the tree ring width information to annual BAI (cm^2^ year^−1^) using the bai.out function in the dplR package [45].

### (c) Population-specific growth rates and its spatial and temporal stability

We analysed growth over the period 1990−2020. For each population and year, we estimated the mean annual growth rate (BAI) and the yearly among-tree variation in growth rates, from here on referred to as spatial stability. The yearly spatial stability was estimated as the mean growth rate divided by the standard deviation in growth rate of the population. Based on this, we next estimated for each population our three response variables: (i) the average annual growth rate; (ii) the temporal stability in annual growth rates, estimated as the average annual growth rate (i) divided by the through-time standard deviation in growth rate (as per [14,46,47]); and (iii) the average spatial stability in growth rates (table 1).

### (d) Age of the trees of the populations

As the growth rate of oaks may change depending on tree age [42], we used the collected tree core samples to determine the age of the oak trees of the specific stands, and used this information as a covariate to control for potential age effects in the statistical analyses. Here, we estimated both the average age of the population and the within-population variation in tree age (CV) in the year 2005 (i.e. the midpoint of the 1990−2020 time period).

### (e) Estimating within-population asynchrony in growth responses

We estimated the degree of within-population tree growth asynchrony, using the dendrochronological method as described in [14,48], using the dplR package in R [45]. This method estimates the temporal growth asynchrony among individuals within a population. Here, we used the annual BAI of each tree for its temporal growth trends. Finally, we estimated the average pairwise Pearson’s correlation coefficients (*r*) of the growth trends for each population, known as rbar in dendrochronology. From this, we then estimated the asynchrony in growth trends as (1 − rbar) as per Li & He [14].

### (f) Among individual genetic variation

To assess the magnitude of inter-individual genetic differentiation within each oak population, we used the leaves collected as above for genotyping with restriction-site associated DNA sequencing. The samples constitute a subset of the samples processed in Hall *et al.* [43]. In brief, we extracted and digested the DNA using DNeasy plant pro kit (Qiagen, Hilden) and HF EcoRI restriction enzyme (NewEngland Biolabs, Ipswich), following the manufacturer’s instructions. The samples were sequenced using Illumina NovaSeq6000 at the Science for Life Laboratory (Sweden), and the raw reads were demultiplexed before delivery. Following that, we filtered and aligned the reads against the reference genome (GenBank assembly: GCA_932294425.1) using *Stacks: process_radtags* and *bwa_mem*, and the contigs were merged and assembled using Stacks: gstacks (Stacks: [49], bwa_mem [50]). To generate a dataset of filtered SNPs, we applied Stacks: populations with the following settings: minimum minor allele frequency of 0.05 and a maximum observed heterozygosity of 0.7, both applied to the metapopulation, a minimum of 80% of the individuals required to be represented at each specific locus for it to be processed for the entire population. Finally, we allowed for multiple SNPs present within a locus, as this study concerns the degree of genetic variability of populations, and a higher number of SNPs provide a more robust estimate of the genetic variability of the population. The resulting SNP dataset was used to determine the population-specific genetic variability of the *Q. robur* populations. This was done by creating a manhattan-based distance matrix using the *as.matrix* function in adegenet, which we then used to quantify the degree of inter-individual genetic variability within the populations using the *betadisper* function in the vegan package [51] in R [52]. The resulting population-specific average distance from the centroid was used in the following analyses as an estimate of the population-specific among-individual genetic variability. We chose to use this measure as our hypothesis concerned consequences of whether individuals within populations were genetically similar or dissimilar [53,54]. This aspect is not specifically captured by traditional population genetic diversity measures (e.g. observed and expected heterozygosity or allelic richness). Finally, to examine whether the genetic variability in ‘adaptive loci’ of populations influenced growth patterns, we used previously attained information on loci identified as being under putative selection [43] to create a dataset consisting only of ‘adaptive SNPs’. From this, we obtained population-specific genetic variability estimates for the putatively adaptive loci.

### (g) Statistical analysis

We performed all statistical analyses in R v. 4.4.1 and RStudio v. 2024.04.2 [52,55]. Significance of fixed effects was evaluated using type III sums of squares (car package [56]).

Using linear regressions with the *lm* function in R, we examined: (i) whether the average annual *growth rate of the populations* was associated with the degree of genetic variability of the oak populations—this model also included the average age of the trees within the stand as an additional explanatory variable to control for age related changes in growth rates; (ii) whether the *temporal stability in growth* was associated with the genetic variability of the population—this model also included age as an additional explanatory variable as among year variation in growth rates may be influenced by the age of the stand due to age-dependent differences in reproductive allocation; and (iii) whether *the spatial stability* in growth was associated with the genetic variability of the population—this model also included the variation in tree age (CV) within the stand as this may impact the variance of growth rates within the stand. Finally, we examined whether the *within-population asynchrony* in growth trends was associated with the genetic variability of the population; and whether the temporal stability in growth of the population increased with within-population asynchrony in growth responses. To estimate the potential importance of variability in adaptive loci (rather than overall genetic variability), we reran the above analyses but this time using the estimated genetic variability for the putatively adaptive loci.

## 3. Results

### (a) No relationship between genetic variability and stand-specific growth rates

**Figure 2.**
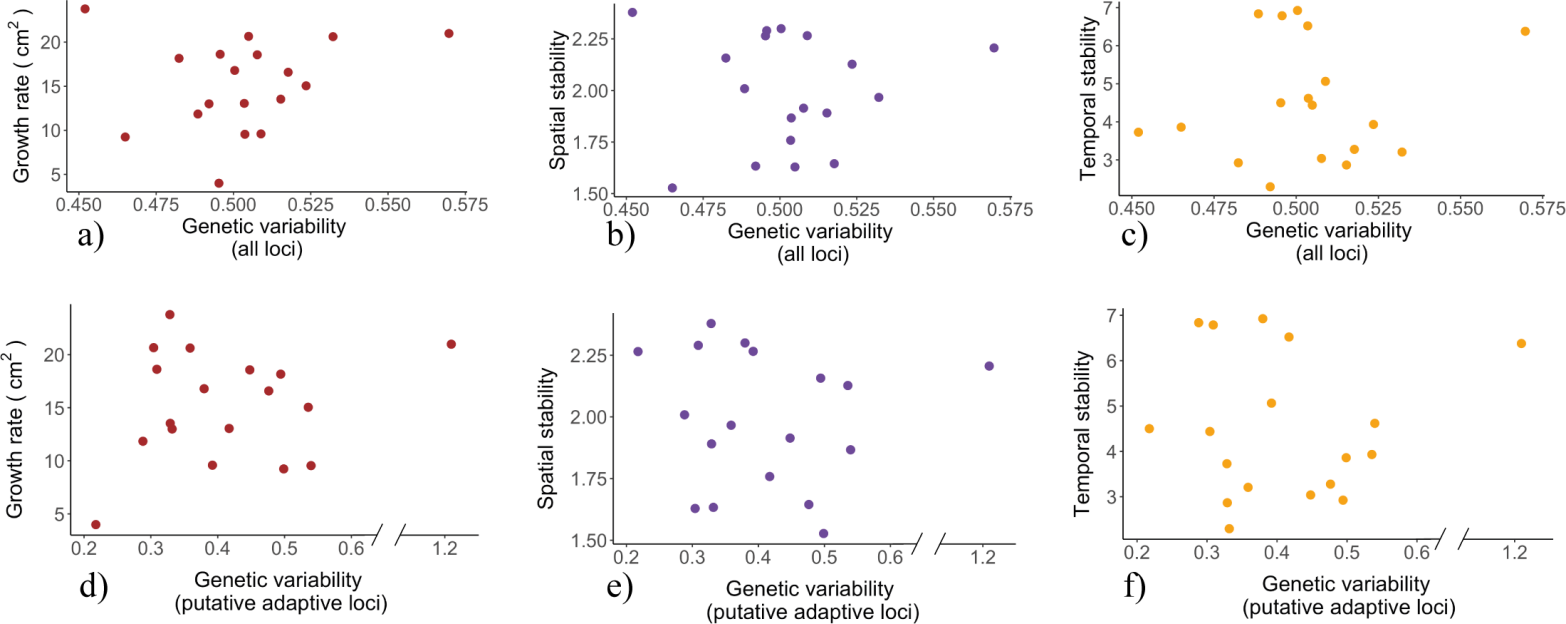
(a) There was no relationship among populations between the average annual growth rate (BAI) of the population and its genetic variability. (b) The temporal stability (among-year variation) in growth rates of the population was not associated with the population’s genetic variability. (c) The spatial stability (among-individual variation) in annual growth rates of a population was not associated with its genetic variability. (d–f) No relationship between the growth rate (d), temporal stability (e) and spatial stability (f), and the populations genetic variability in putatively adaptive loci. Each dot represents the average growth estimate (growth rate, temporal stability spatial stability) and the genetic variability of the population. Note the break on the *x*-axis for the genetic variability in putative adaptive loci*.*

The overall growth rate of the population was not associated with the inter-individual genetic variability of the oak population, but with a trend of lower growth rates with increasing average age of the trees of the stand (genetic variability: *F*_1,15_ = 0.08, *p* = 0.77; average age: *F*_1,15_ = 3.47, *p* = 0.082) (figure 2a). There was also no relationship between the genetic variability in the ‘adaptive loci’ and the populations overall growth rate (genetic variability: *F*_1,15_ = 0.41, *p* = 0.53; average age: *F*_1,15_ = 3.16, *p* = 0.096) (figure 2d). These results (here and below) also remained qualitatively similar when the potential outlier population with the highest genetic variability was excluded from the analyses, and when stand basal area—a common proxy for stand density and competition—was included as an additional covariate.

### (b) No relationship between genetic variability and spatial or temporal stability in growth rates

We found no relationship between the temporal stability in growth rates and the genetic variability or the average age of the population (genetic variability: *F*_1,15_ = 0.58, *p* = 0.46; average age of the trees: *F*_1,15_ = 0.66, *p* = 0.43) (figure 2b). The spatial stability (among-individual variation) in growth rates of the oak population was also not associated with its genetic variability or the variation in tree age (genetic variability: *F*_1,15_ = 0.01, *p* = 0.91; CV of tree age: *F*_1,15_ = 1.41, *p* = 0.25) (figure 2c). Finally, there was no relationship between the genetic variability in the ‘adaptive loci’ and the populations overall temporal (genetic variability: *F*_1,15_ = 0.79, *p* = 0.39; average age of the trees: *F*_1,15_ = 0.74, *p* = 0.40) or spatial stability (genetic variability: *F*_1,15_ = 0.04, *p* = 0.85; CV of tree age: *F*_1,15_ = 1.38, *p* = 0.26) (figure 2e,f).

### (c) Temporal stability of population growth increased with increasing among-individual asynchrony

**Figure 3.**
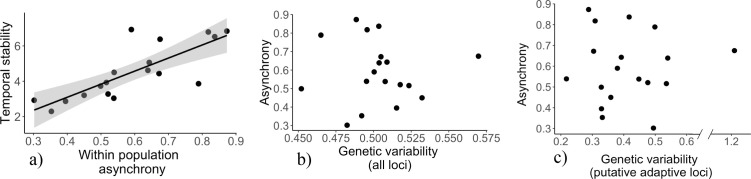
(a) The temporal stability of population growth rates increased with increasing asynchrony in growth trends of its individuals, (b) however, the degree of asynchrony among individuals within a population was unrelated to the populations genetic variability and (c) unrelated to the populations genetic variability in putative adaptive loci. Each dot represents a population, and the presence of a line signifies a statistically significant relationship (predicted linear relationship with a 95% confidence interval). Note the break on the *x*-axis for the genetic variability in putative adaptive loci.

The temporal stability in growth rates of the populations increased with increasing among-individual asynchrony in growth (*F*_1,16_ = 26.96, *p* < 0.001; figure 3a). However, the asynchrony in growth responses among individuals within the population was independent of its genetic variability (*F*_1,16_= 0.02, *p* = 0.88; figure 3b) and of the genetic variability in its ‘adaptive loci’ (*F*_1,16_= 0.09, *p* = 0.77; figure 3c).

## 4. Discussion

Theory, field experiments and observational studies on the performance of populations, species and ecosystems have highlighted that increased intraspecific diversity may promote overall productivity and increase the temporal stability of productivity. However, the results in this study, based on a combination of long-term data on individual growth trends and population genetics for 18 naturally occurring populations of *Q. robur*, do not support the hypothesis that higher genetic variability of populations should increase the average growth rate of the population, its temporal stability, spatial stability and be related to the degree of asynchrony in growth rates. However, we found that the temporal stability of the populations increased with increasing asynchrony of growth responses among individual trees. This disconnect between the genetic variability of the population and the different aspects of tree growth points towards a negligible or small role of genetic variation in structuring productivity patterns in natural populations of oaks and instead implicate phenotypic plasticity and epigenetic effects as potentially important sources of variation in tree growth.

The conclusion that genetic diversity does not impact oak growth rates is in sharp contrast to the increased performance in populations with higher intraspecific diversity documented in a wide variety of taxa, including arthropods, insects, birds and fish [2,3,7,17], and earlier findings on the effects of genotype diversity in plants [16,18,57–59]. Instead, our findings align with previous experimental research suggesting no or negligible effects of genetic diversity on productivity in tree species [29,30,32] (but see [31]). Fischer *et al.* [29] and Bongers *et al.* [30] examined the effects of genetic variation in experimental forest plots by manipulating the number of genotypes [29] or seed families [30], and found no effects of genetic variation on the overall productivity. Tang *et al.* [32], using the same field experiment as Bongers *et al.* [30], reported no direct effect of genetic diversity on productivity—but possible indirect effects mediated via herbivory and soil fungal community which impacted community productivity. However, the stands in these studies were young—Fischer *et al.* [29] planted in 2006/2007, while Bongers *et al.* [30] and Tang *et al.* [32] were planted in 2009/2010. Any complementarity effects of genetic diversity that might occur after canopy closure—which is where the complementary effects of species diversity are largest in trees [26,27]—may therefore have gone undetected in the experimental plots. This conclusion is supported by [31] who reported on a long-term (43 years) provenance mixing experiment on Norway spruce (*Picea abies*), showing that inter-provenance complementarity reduced inter-individual competition and increased stem diameter growth as well as productivity at the stand level. Here, we show that the expected positive effects of genotype diversity on the average growth rate and the temporal and spatial stability of tree productivity do not manifest in natural populations with a closed canopy forest, despite being exposed to complex conditions where the benefits of diversity are typically strongest [17].

The absence of positive effects on tree growth from genetic diversity in our study system may stem from the natural history of oak, and many other tree species. Trees in general and oaks in particular exhibit high gene flow among populations due to wind pollination, large intra-population variation compared with other plants and relatively low genetic differentiation among populations [19,21]. Conclusions based on manipulation experiments of relatively few genotypes may therefore not inform about the role of genetic variation in naturally occurring tree populations. With regard to the level of diversity, a meta-analysis by Raffard *et al.* [7] reported a saturating curve with increasing levels of genetic diversity, suggesting limited effects of increased diversity at higher richness levels due to functional redundancy [7]. This suggests that species with genetically diverse populations, such as oak, may have limited benefits of higher levels of diversity in naturally occurring populations. However, the asymptotic increase indicated by Raffard *et al.* [7] is not universal. Forsman & Wennersten [3] found that for 10 out of 12 experimental studies, population performance increased linearly with genetic diversity. This supports that the effects of increasing diversity levels are context and species-specific. One additional explanation for the lack of positive effects may be that local adaptations and coadapted gene complexes are disrupted by non-local gene variants and admixture brought about by high gene flow due to wind pollination. Gene flow may therefore increase intra-population diversity without bringing diversity-driven benefits that outweigh potential negative impacts of outbreeding [3].

With regard to the temporal stability of growth, our results suggest that the level of genetic diversity in the *Q. robur* populations that we investigated was insufficient to buffer against between-year fluctuations in environmental conditions. We are not aware of any previous studies that have explicitly examined the role of genetic diversity for the temporal stability in productivity of tree populations, limiting comparisons. However, previous research reported clear positive effects of tree species richness on the temporal stability in productivity, which was attributed to increased asynchrony in growth trends and overyielding [28,47,60]. That the importance of genetic diversity seems to differ from that of species diversity may reflect that only about 25% of the total trait variation in woody plant communities is attributed to intraspecific variation [61]. Finally, it is possible that we have underestimated any positive effects from increased functional diversity or from diversity in specific genes and traits. Yet, that there were no significant associations even with the genetic variation in the putatively adaptive loci argues against this interpretation.

This study identified one aspect of intraspecific diversity of importance for tree growth, as the temporal stability of growth increased with increasing asynchrony in growth trends among individuals within the populations. This finding is consistent with Li & He [14] who, using a global dendrochronological dataset, reported that the temporal stability in growth rates of the populations increased with increasing within-population tree-growth asynchrony. That varying responses of different phenotypes to changing environmental conditions promote population performance is also in agreement with expectations from theory [2,3,5,62]. The apparent disconnect in this study between the genetic diversity and the among-individual growth asynchrony of the populations suggest that the growth trajectories of individual trees may be influenced both by heterogeneous environmental conditions (e.g. soil nutrients, shade and hydrology) [28,63,64] and by phenotypic differences in traits such as phenology, physiology, reproductive allocation, disease- or pathogen-induced stress [33,36–38,65,66,67] which may not fully be reflected by the estimated genetic diversity of the populations.

In conclusion, we found no effects of genetic variability on the overall annual growth rate, temporal stability, spatial stability or the among-individual asynchrony of growth rates in the natural oak populations that we investigated. This is in sharp contrast to several previous studies in other taxa; possibly due to the nature and life history of naturally occurring tree populations, with long lifespans, high gene flow and substantial within-population genetic and phenotypic variation. As expected, greater among-individual asynchrony of growth responses within populations was associated with higher temporal stability. Identifying which environmental factors and phenotypic traits contribute to asynchronous growth responses is an important next step towards improving our mechanistic understanding of temporal stability of tree growth and forest productivity.

## Data Availability

The data and R code used for the statistical analysis is available at [68].
